# Supplementary material for: IRF8 Regulates Intrinsic Ferroptosis through Repressing p53 Expression to Maintain Tumor Cell Sensitivity to Cytotoxic T Lymphocytes
Source: Cells. 2023 Jan 13;12(2):310. doi: 10.3390/cells12020310 (PMC9856547; doi:10.3390/cells12020310)
Supplement: Supplementary file 1 [file cells-12-00310-s001.zip › cells-2133528-supplementary.pdf]

Supplemental Data

Sequence name / optimized for

mIRF8

ORF	Protected sites	Protected areas	Motifs to avoid
13-1290 [ATG...TGA]	1-6 SalI [GTCGAC]<br>1291-1296 EcoRI [GAATTC]		SalI [GTCGAC]<br>EcoRI [GAATTC]

```
                    M  C  D  R  N  G  G  R  R  L  R  Q  W  L  I  E  Q  I  D
1.    GTCGACGCCACCATGTGCGATAGAAATGGTGGCAGACGGCTGCGGCAGTGGCTGATCGAGCAGATCGAT
      S  S  M  Y  P  G  L  I  W  E  N  D  E  K  T  M  F  R  I  P  W  K  H
70.   AGCAGCATGTACCCCGGCCTGATCTGGGAGAACGACGAGAAAACAATGTTCAGGATCCCCTGGAAGCAC
      A  G  K  Q  D  Y  N  Q  E  V  D  A  S  I  F  K  A  W  A  V  F  K  G
139.  GCCGGCAAGCAGGACTACAATCAAGAGGTGGACGCCAGCATCTTCAAGGCCTGGGCCGTGTTCAAGGGC
      K  F  K  E  G  D  K  A  E  P  A  T  W  K  T  R  L  R  C  A  L  N  K
208.  AAGTTCAAAGAGGGCGACAAGGCCGAGCCTGCCACCTGGAAAACCAGACTGAGATGCGCCCTGAACAAG
      S  P  D  F  E  E  V  T  D  R  S  Q  L  D  I  S  E  P  Y  K  V  Y  R
277.  AGCCCCGACTTCGAGGAAGTGACCGACAGAAGCCAGCTGGACATCAGCGAGCCCTACAAGGTGTACCGG
      I  V  P  E  E  E  Q  K  C  K  L  G  V  A  P  A  G  C  M  S  E  V  P
346.  ATCGTGCCCGAAGAGGAACAGAAATGCAAGCTGGGAGTTGCCCCTGCCGGCTGTATGTCTGAAGTGCCC
      E  M  E  C  G  R  S  E  I  E  E  L  I  K  E  P  S  V  D  E  Y  M  G
415.  GAAATGGAATGCGGCAGAAGCGAGATCGAGGAACTGATCAAAGAACCCAGCGTGGACGAGTACATGGGC
      M  T  K  R  S  P  S  P  P  E  A  C  R  S  Q  I  L  P  D  W  W  V  Q
484.  ATGACCAAGCGGAGCCCATCTCCTCCAGAGGCCTGCAGATCTCAGATCCTGCCTGATTGGTGGGTGCAG
      Q  P  S  A  G  L  P  L  V  T  G  Y  A  A  Y  D  T  H  H  S  A  F  S
553.  CAGCCTTCTGCTGGACTGCCACTGGTTACAGGCTACGCCGCCTACGATACACACCACAGCGCCTTCAGC
      Q  M  V  I  S  F  Y  Y  G  G  K  L  V  G  Q  A  T  T  T  C  L  E  G
622.  CAGATGGTCATCTCCTTCTACTACGGCGGCAAGCTCGTTGGCCAGGCCACCACAACATGTCTGGAAGGC
      C  R  L  S  L  S  Q  P  G  L  P  K  L  Y  G  P  D  G  L  E  P  V  C
691.  TGTCGGCTGAGCCTGTCTCAACCTGGCCTGCCTAAGCTGTACGGCCCCGATGGACTGGAACCTGTGTGT
      F  P  T  A  D  T  I  P  S  E  R  Q  R  Q  V  T  R  K  L  F  G  H  L
760.  TTCCCTACCGCCGACACAATCCCCAGCGAGAGACAGAGACAAGTGACCCGGAAGCTGTTCGGACACCTG
      E  R  G  V  L  L  H  S  N  R  K  G  V  F  V  K  R  L  C  Q  G  R  V
829.  GAACGGGGAGTGCTGCTGCATAGCAACAGAAAGGGCGTGTTCGTGAAGCGGCTGTGCCAGGGCAGAGTG
      F  C  S  G  N  A  V  V  C  K  G  R  P  N  K  L  E  R  D  E  V  V  Q
898.  TTCTGTTCTGGCAATGCCGTCGTGTGCAAAGGCAGACCCAACAAGCTGGAACGCGACGAGGTGGTCCAG
      V  F  D  T  N  Q  F  I  R  E  L  Q  Q  F  Y  A  T  Q  S  R  L  P  D
967.  GTGTTCGACACCAACCAGTTCATCAGAGAGCTGCAGCAGTTCTACGCCACACAGAGCAGACTGCCCGAC
      S  R  V  V  L  C  F  G  E  E  F  P  D  T  V  P  L  R  S  K  L  I  L
1036. AGCAGAGTGGTGCTGTGCTTCGGCGAAGAGTTCCCCGATACCGTGCCTCTGAGAAGCAAGCTGATCCTG
      V  Q  V  E  Q  L  Y  A  R  Q  L  V  E  E  A  G  K  S  C  G  A  G  S
1105. GTGCAGGTCGAGCAGCTGTACGCCAGACAGCTGGTGGAAGAGGCCGGAAAGTCTTGTGGCGCCGGATCT
      L  M  P  A  L  E  E  P  Q  P  D  Q  A  F  R  M  F  P  D  I  C  T  S
1174. CTGATGCCCGCTCTGGAAGAACCTCAGCCTGACCAGGCCTTCAGGATGTTCCCTGACATCTGCACCAGC
      H  Q  R  P  F  F  R  E  N  Q  Q  I  T  V  *  *
1243. CACCAGCGGCCATTCTTCAGAGAGAACCAGCAGATCACCGTGTGATGAGAATTC
```

Figure S1. Mouse IRF8 codon usage optimized cDNA sequence. The two highlighted sequences at the beginning and end indicate the restriction enzyme Sal1 and EcoR1, respectively. The protein amino acid sequence is listed under the DNA sequence.

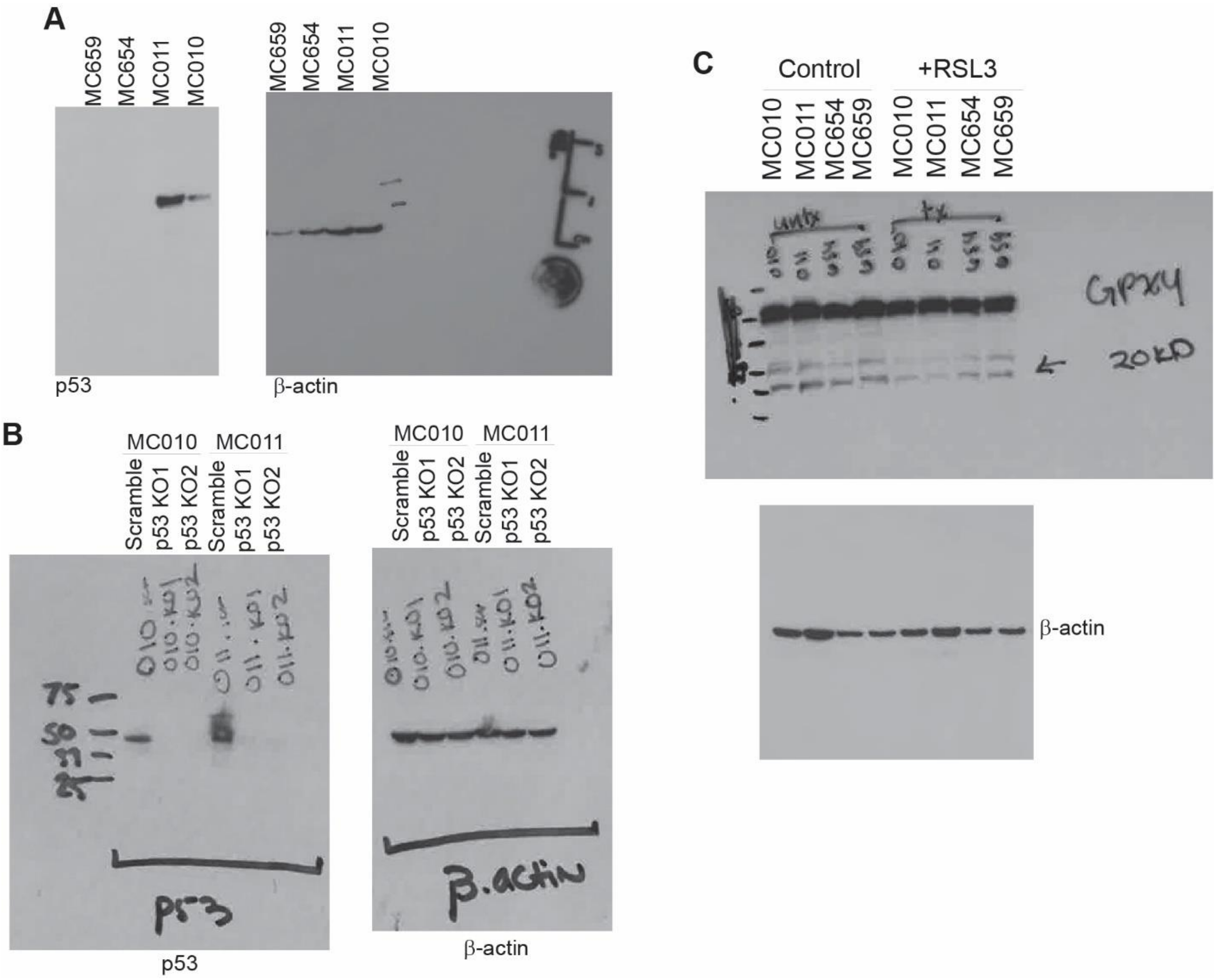

Figure S2. Western blots. A. Original Western blots for Figure 2E. **B**. Original Western blots for Figure 3C. **C**. Original Western for Figure S3.

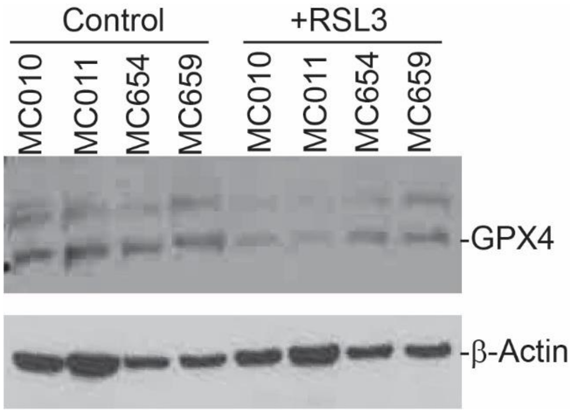

Figure S3. GPX4 protein level in tumor cells. The indicated WT and IRF8 KO cell lines were cultured in the absence (Control) or presence of RSL3 for approximately 24h. The cells analyzed by Western for GPX4 protein level. β-action was used as normalization control.

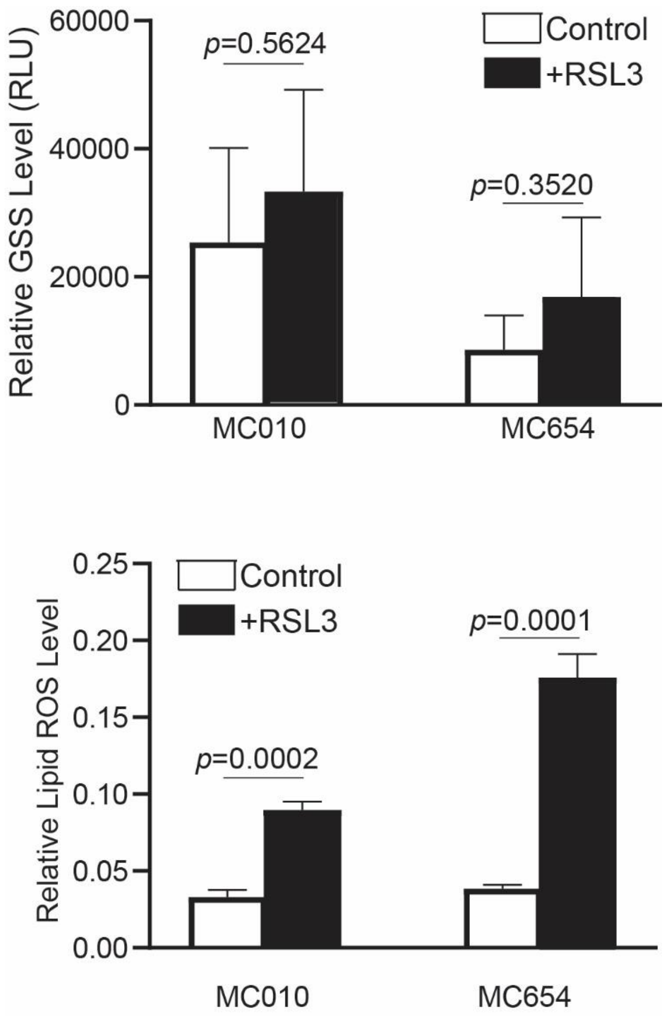

Figure S4. GSH and lipid ROS level in tumor cells. A. The pair of WT (MC654) and IRF8 KO (MC010) tumor cell lines were cultured in the absence (Control) or presence of RSL3 for 3h. Cells were then analyzed for GSH level using the GSH luminescent-based assay. GSH level is presented as relative light units (RLU) according to the manufacturer's instruction. **B**. Cells were treated as in A for 3h, incubated with BODIPY 581/591 C11 lipid peroxidation sensor for 30 min. Cells were then analyzed with a flow cytometry. The lipid ROS level is presented as the ratio of mean FL1 intensity/mean FL2 intensity.
